# Mutual maintenance of di- and triploid *Pelophylax esculentus* hybrids in R-E systems: results from artificial crossings experiments

**DOI:** 10.1186/s12862-017-1063-3

**Published:** 2017-10-17

**Authors:** Dmitry Dedukh, Spartak Litvinchuk, Juriy Rosanov, Dmitry Shabanov, Alla Krasikova

**Affiliations:** 1 Saint-Petersburg State University, Saint-Petersburg, Russia; 2 Institute of Cytology, Russian Academy of Sciences, Saint-Petersburg, Russia; 3 V.N. Karazin Kharkiv National University, Kharkiv, Ukraine; 4 Saint-Petersburg State University, 7-9, Universitetskaya nab, 199034 Saint-Petersburg, Russia

**Keywords:** Hybrid population systems, Polyploid hybrid, Gamete, Genome elimination, Karyotype, Hemiclonal reproduction

## Abstract

**Background:**

Interspecies animal hybrids can employ clonal or hemiclonal reproduction modes where one or all parental genomes are transmitted to the progeny without recombination. Nevertheless, some interspecies hybrids retain strong connection with the parental species needed for successful reproduction. Appearance of polyploid hybrid animals may play an important role in the substitution of parental species and in the speciation process.

**Results:**

To establish the mechanisms that enable parental species, diploid and polyploid hybrids coexist we have performed artificial crossing experiments of water frogs of *Pelophylax esculentus* complex. We identified tadpole karyotypes and oocyte genome composition in all females involved in the crossings. The majority of diploid and triploid hybrid frogs produced oocytes with 13 bivalents leading to haploid gametes with the same genome as parental species hybrids usually coexist with. After fertilization of such gametes only diploid animals appeared. Oocytes with 26 bivalents produced by some diploid hybrid frogs lead to diploid gametes, which give rise to triploid hybrids after fertilization. In gonads of all diploid and triploid hybrid tadpoles we found DAPI-positive micronuclei (nucleus-like bodies) involved in selective genome elimination. Hybrid male and female individuals produced tadpoles with variable karyotype and ploidy even in one crossing owing to gametes with various genome composition.

**Conclusions:**

We propose a model of diploid and triploid hybrid frog reproduction in R-E population systems. Triploid *Pelophylax esculentus* hybrids can transmit genome of parental species they coexist with by producing haploid gametes with the same genome composition. Triploid hybrids cannot produce triploid individuals after crossings with each other and depend on diploid hybrid females producing diploid eggs. In contrast to other population systems, the majority of diploid and triploid hybrid females unexpectedly produced gametes with the same genome as parental species hybrids coexist with.

**Electronic supplementary material:**

The online version of this article (10.1186/s12862-017-1063-3) contains supplementary material, which is available to authorized users.

## Background

Interspecies hybridization is currently considered to play a significant role in the speciation processes not only in plants but also among invertebrates and lower vertebrates [1, 2]. Animal interspecies hybrids can develop clonal or hemiclonal reproduction modes where one or all parental genomes are transmitted to progeny without recombination [3, 4]. Some interspecies hybrids need to regularly cross back with parental species for successful reproduction [3, 4]. Absence of recombination and inability to reproduce independently are thought to prevent long-term evolutionary success [5]. Appearance of polyploid animals can solve these evolutionary problems since clonal genome can in fact recombine [6, 7]. Moreover, polyploid hybrids can generate independent species [1, 6, 8–10]. The role of the polyploid hybrids as intermediate stage towards independent species remains poorly understood. It is not known how polyploid and diploid interspecies animal hybrids appear and are maintained in the majority of population systems of clonally and hemiclonally reproducing species.

To study interspecies hybridization, hemiclonal reproduction and maintenance of di- and polyploid hybrid lines we focused on the European water frog (the *Pelophylax esculentus* complex) model system. The complex includes two parental species, *P. ridibundus* (RR genotype, 2n = 26) and *P. lessonae* (LL genotype, 2n = 26), which after crossing, produce hybridogenetic frog *P. esculentus* (RL genotype, 2n = 26) [11, 12].

During hybridogenetic way of reproduction the gametogenesis of diploid *P. esculentus* leads to the elimination of the genome of one parental species, while the genome of the other parental species is duplicated and transferred to the gametes [12, 13]. For the maintenance of hybrid frogs their gametogenesis changes depending on the parental species they coexist with. When diploid hybrids coexist with *P. lessonae* they usually produce gametes with *P. ridibundus* genome and vice versa [13–16]. Additionally, two forms of triploid (RRL and LLR, 3n = 39) and even tetraploid (RRLL, 4n = 48) hybrid frogs exist in natural population systems [7, 13, 16–19].

Coexistence between different forms of hybrid frogs and one or both parental species gives rise to various population systems. Widespread and well-studied population systems are represented by only diploid or diploid and triploid *P. esculentus* coexisting with *P. lessonae* (L-E system) as well as pure hybrid population systems where diploid hybrids coexist with triploids without the parental species (E system) [13, 16–18, 20, 21]. Population systems where di- and triploid hybrid frogs coexist with *P. ridibundus* (R-E system) occasionally occur in central Europe and are extremely abundant in the Eastern Ukraine [13, 15, 16, 22].

Triploid hybrids are widespread in population systems of not only  *P. esculentus* complex but also other hybrid complexes [3, 4, 10, 23]. Triploid hybrids usually produce haploid gametes with those genomes that they carry in two copies while genome represented by a single copy is eliminated [3, 7, 9, 24]. As expected, the majority of triploid *P. esculentus * hybrids produce haploid oocytes with 13 bivalents corresponding to the genome that they carry in two copies [25]. In the present work, we investigate whether oocytes with 13 bivalents generated by triploid hybrid *P. esculentus* females can produce haploid gametes and give rise to diploid *P. ridibundus* or *P. esculentus* animals.

Unreduced eggs produced by diploid *P. esculentus* females reveal lack of genome elimination and additional endoreplication events [17–19, 25]. Such eggs are thought to be important to the emergence of triploid individuals [13–15]. Nevertheless, in the classical studies on hybrid frogs correlation between eggs size and their ploidy was estimated by indirect method – via measurement of their size [14, 26, 27]. At the same time, the analysis of lampbrush chromosomes that appear in growing oocytes represent reliable and direct method for identification of genomes transmitted in the hybrid oocytes [25, 28, 29]. In our previous work we have observed that diploid *P. esculentus* females can produce oocytes with 26 bivalents corresponding to the genomes of both parental species (RL oocytes) [25]. Thus, our other task was to check if oocytes with 26 bivalents can give rise to triploid animals.

It should be stressed that deviations in genome elimination during hybrid frog gametogenesis can lead to the formation of gametes with various genome compositions. Individual hybrid animals producing gametes with variable genome composition were found in *P. esculentus* population systems [7, 17, 30]. Moreover, our previous results demonstrated that individual di- and triploid hybrid frogs can produce oocytes with variable ploidy [25]. We thus aimed to check if such oocytes participate in the appearance of genomically variable progeny.

Previously, the genome composition of tadpole cells was inferred after morphological examination of froglets, protein electrophoresis, measurements of erythrocytes and karyotyping without precise genome identification [14, 16, 26]. Microsatellite analysis was previously successfully carried out for reliable determination of tadpoles karyotypes obtained after crossing frogs from Denmark and Sweden. Nevertheless, microsatellites remain quite variable in various population systems [7, 17–19]. Cytogenetic analysis reveals a promising method for parental species identification, but still suffers from the absence of reliable markers that are suitable for individual identification from various populations [28, 29, 31–34]. In our previous work we have found species-specific marker using fluorescent in situ hybridization: chromosome bearing nucleolus organizer region (NOR) differs in the number of interstitial (TTAGGG)_n_ sites in karyotypes of parental species (*P. ridibundus* and *P. lessonae*) [28]. *P. lessonae* nucleolar chromosome bears one interstitial telomeric site while *P. ridibundus* nucleolar chromosome bears two interstitial telomeric sites [28].

Abnormalities in gonadal morphology and gamete formation were observed among some adult *P. esculentus* but little is known about the presence of germline cells in the gonads of hybrid tadpoles [30, 35–37]. Ogielska observed micronuclei in the gonads of hybrid tadpoles that were suggested to be involved in the selective genome elimination [38]. The role and appearance of the micronuclei are still vague and they were not studied in hybrids from other population types [16]. Thus, our additional aim was to check the presence of germ cells and micronuclei within the gonads of hybrid tadpoles.

In order to answer these questions, we have identified karyotypes of tadpoles appeared after artificial crosses, determined chromosomal sets transmitted in the oocytes of all females participated in the crossings and assessed the presence of germ cells in the gonads of hybrid tadpoles. We suggest a scheme for diploid and triploid hybrid frog maintenance in the studied R-E system.

## Methods

### Frog sampling

All European water frogs were collected from the drainage area of the Seversky Donets River in the Kharkiv region (Eastern Ukraine). 10 *P. ridibundus* individuals, 25 diploid, 15 triploid hybrids with RRL genotype and 2 triploid hybrids with LLR genotype in the R-E systems were captured. Parental species and all hybrids, except triploid frogs with LLR genotype, were represented by both sexes; while triploid frogs with LLR genotype were represented by female individuals only (Additional file 1: Table S1). Animals were caught during two breeding seasons in 2013 and 2014. All manipulations with animals were carried out in accordance with the national and international guidelines. Field studies did not involve endangered or protected species. All specimen were collected in the regions of Ukraine not considered protected areas, thus no specific permissions were required for work on those locations. Techniques used in the capture, breeding, tissue sampling and euthanasia sought to minimize animal suffering. Each individual was anaesthetized by methoxyethane or submersion in a 1% solution of 3-aminobenzoic acid ethyl ester (MS 222). All procedures were approved by the Local Animal Ethic Committee of Saint-Petersburg State University (# 131–03-3).

### DNA flow cytometry

We have performed DNA flow cytometry genome composition identification of all collected adult frogs [22, 30]. Given the difference in the genome sizes between parental species it is possible to distinguish between parental species (2n = 16.00 ± 0.35 pg for *P. ridibundus* and 2n =14.00 ± 0.35 pg for *P. lessonae*) and different forms of hybrid frogs (2n = 14.90 ± 0.35 pg for diploid *P*. *esculentus*; 3n =21.80 ± 0.35 for LLR hybrids and 3n = 22.9 ± 0.35 for RRL hybrids) [22, 30]. Blood from the femoral vein taken from anaesthetized (0.15% MS222 (Sigma)) animals was diluted with the solution of 0.1% Triton X100, 20 μg/ml ethidium bromide and 15 mM MgCl_2_ and measured in flow cytometer constructed at the Institute of Cytology, Russian Academy of Sciences, St. Petersburg. Obtained results were compared with two reference standards: blood of grass frog (*Rana temporaria* Linnaeus, 1758) and male domestic mouse (*Mus musculus* Linnaeus, 1758; spleenocytes, C57B1 line) [22]. Histograms of cellular DNA composition were created using the formula: DNA content = (samples mean peak)/(reference standard peak) × (reference standard genome size).

### Crossing experiments

Pairs of frogs were sequestered in tanks measuring 60 сm × 40 сm × 30 сm, with 23 pairs in total (Additional file 1: Table S1). Individuals from the same crossings are named identically except “m” and “f” letter indicating male or female correspondingly (Additional file 1: Table S1). To stimulate spawning, males and females were injected with 400 μl of Surfagon (synthetic analog of luteinizing hormone) at 5 μg/ml [39]. Females spawned after 12–48 h. Eggs were placed in separate tanks filled with 5–7 сm of water and aerated using compressor. Stages of tadpoles development were identified according to Gosner [40]. Starting from stage 28 (hind limb bud development) to stage 42 (forelimbs emergence), every week we randomly caught 5–20 tadpoles from each clutch. Tadpoles were placed in anesthetic solution (0.15% MS 222) where gills, intestine and tail were dissected and stored in ethanol:glacial acetic acid 3:1 solution.

### Lampbrush and metaphase chromosomes preparation

After spawning, all females were used for lampbrush chromosome analysis. Before ovary dissection frogs were anaesthetized with 0.15% MS 222 solution. Lampbrush chromosomes were obtained from the oocyte nuclei following standard procedures, described by Callan et al. [41] with modifications suggested by Gall et al. [42]. Lampbrush chromosomal spreads were dehydrated in a series of ethanol solutions – 50%, 70%, 96% – and air dried.

To obtain metaphase chromosomes, organs dissected from tadpoles and fixed in ethanol:glacial acetic acid 3:1 solution were dissected into fragments. The fragments were then placed in 70% acetic acid for 5 min. Cell suspension was transferred dropwise onto slides heated to 60 °С.

### Fluorescence in situ hybridization

DNA/DNA FISH with a probe to (TTAGGG)_n_ repeat was performed on the lampbrush and metaphase chromosomes as described elsewhere [25, 28]. Metaphase chromosomes were pretreated with RNAse A (100–200 μg/ml) for 1 h and pepsin (0.01% in 0.01 N HCl) for 10 min, then postfixed in 2% paraformaldehyde solution (PBS, 50 mM MgCl_2_) for 10 min. Lampbrush chromosomes were not pre-treated before FISH. Hybridization mix contained 40% formamide, 2 × SSC (saline-sodium citrate buffer; 20 × SSC – 3 M NaCl 300 мМ Na3C6H5O7), 12% dextrane sulphate, 5 ng/μl single stranded (TTAGGG)_5_ probe conjugated with biotin and 10 to 50-fold excess of tRNA. Hybridization mix was applied to the metaphase and lampbrush chromosome preparations under coverslips. Chromosomes were denatured for 5 min at 82 °C. Hybridization was performed overnight at room temperature. Preparations were then washed in 2 × SSC at 42 °C. Biotin labeled probe was detected by avidin conjugated with Cy3 dye (Jackson ImmunoResearch Laboratories). The slides were then counterstained in DABCO antifade solution containing 1 mg/ml DAPI. At least 3 full metaphase plates with clearly visible FISH signals were examined to identify tadpoles karyotype. As a control, karyotypes of tadpoles resulting from crosses of two *P. ridibundus* were identified.

### Wide-field microscopy

Leica fluorescence microscope DM 4000В was used for analysis of metaphase and lampbrush chromosomes. Detection of fluorescent signals was performed with appropriate filter cubes (Leica Wetzlar GmbH, Germany). Images were taken by a monochrome digital camera DFC350 FX under 10×, 20×, 40×/1 and 100×/1 objectives using Leica CW 4000 FISH software.

### Confocal laser scanning microscopy

Dissection of gonads from tadpoles was carried out under stereomicroscope Leica MZ16. Tissue was fixed in 2% paraformaldehyde in PBS for 90 min. For long term storage tissue was kept in PBS with 0.02% NaN_3_ added. Prior to microscope examination, tissue was incubated in 0.5% solution of Triton X100 for 1 h, washed in PBS for 15 min and kept in PBS containing 1 mg/ml DAPI overnight. Tissue was placed in a drop of DABCO antifade solution containing 1 mg/ml DAPI and confocal laser scanning microscopy was carried out using Leica TCS SP5 microscope. Specimens were analyzed using HC PL APO 40× objective with 405 nm UV laser. Images were captured using LAS AF software (Leica Microsystems, Germany).

## Results and discussion

### Complex cytogenetic analysis of tadpoles and lampbrush chromosomes represents a relevant approach for investigation of hybrid frogs gametogenesis

**Fig. 1:**
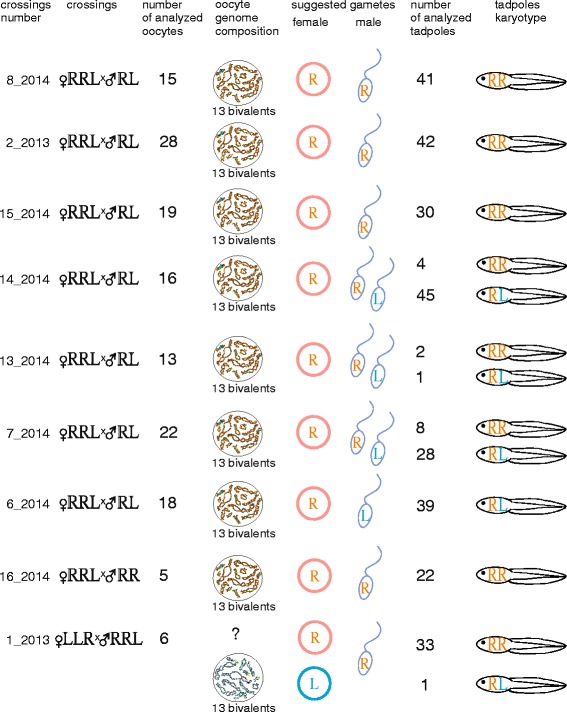
Results of crossing experiments of triploid hybrid females with RRL or LLR genotypes with *P. ridibundus* and di- or triploid hybrid males. Tadpoles resulting from crosses were represented by *P. ridibundus* (RR) and *P. esculentus* (RL) individuals. Triploid hybrid females with RRL genotype produced oocytes with 13 bivalents corresponding to *P. ridibundus* chromosomes (orange). All observed oocytes of triploid hybrid female with LLR genotype included 13 bivalents corresponding to *P. lessonae* chromosomes (blue). Obtained results allow to assume that triploid hybrid females with RRL genotypes produce gametes with *P. ridibundus* genome (R), triploid hybrid females with LLR genotype produce gametes with *P. ridibundus* (R) and *P. lessonae* (L) genomes. Question marks (?) indicate discrepancy between oocyte chromosomal sets and gametes genome composition inferred from crossing experiments. Diploid hybrid males presumably produce haploid sperm with *P. ridibundus* (R) and *P. lessonae* (L) genomes and finally triploid hybrid males produce haploid sperm with *P. ridibundus* genome (R). Crossing numbers correspond to Additional file 1: Table S1

**Fig. 2:**
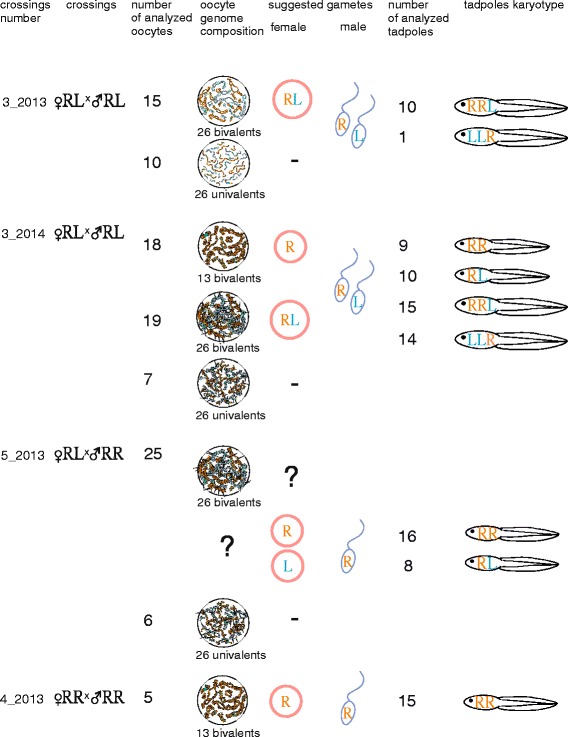
Results of crossings experiments of *P. ridibundus* individuals and diploid hybrid females with *P. ridibundus* and diploid *P. esculentus* males. Tadpoles obtained after crossings were represented by triploid hybrids with RRL and LLR genotypes, *P. ridibundus* (RR) and *P. esculentus* (RL) individuals. Diploid hybrid females produced oocytes with 26 bi- and univalents corresponding to *P. ridibundus* (orange) and *P. lessonae* (blue) chromosomes, 13 bivalents corresponding to *P. ridibundus* (orange) chromosomes. Obtained results allow us to assume that diploid hybrid females produce unreduced gametes (RL) and gametes with *P. ridibundus* (R) or *P. lessonae* (L) genomes. Oocytes with 26 univalents presumably cannot overcome meiosis and give aneuploidy gametes. Diploid hybrid males presumably produce haploid sperm with *P. ridibundus* (R) and *P. lessonae* (L) genomes. Two *P. ridibundus* individuals produce haploid gametes with *P. ridibundus* genome. Question marks (?) indicate discrepancy between oocyte chromosomal sets and gametes genome composition inferred from crossing experiments. Crossing numbers correspond to Additional file 1: Table S1

After genome composition of all mature frogs was identified using DNA flow cytometry we have performed crossings of the hybrid females with *P. ridibundus* individuals and hybrid males. Tadpoles were obtained from 13 crosses: eight crossings of triploid hybrid females of the RRL genotype with diploid hybrid males and a *P. ridibundus* male, one crossing of triploid hybrid female of the LLR genotype with triploid male of the RRL genotype (Fig. 1), three crossings of diploid hybrid females with diploid hybrid males or *P. ridibundus* individuals and finally one crossing between *P. ridibundus* individuals (Fig. 2). 10 attempts were unsuccessful and females did not spawn or spawned without any eggs developing (Additional file 1: Table S1).

During developmental stages 1–18, according to Gosner 1960 [40], the survival levels of eggs and embryos in the majority of clutches produced by hybrid frogs were lower (survival of tadpoles was about 60–70%) compared to the parental species (survival of tadpoles was 89%) (Additional file 2: Table 2). Moreover, four crosses of hybrid females with hybrid males and *P. ridibundus* displayed low levels of survival – from 2% to 20% (Additional file 2: Table 2). Some tadpoles died during early stages of development due to abnormal cleavage, exogastrulation, protruding of yolk and liquid-filled bodies.

Using FISH with (TTAGGG)_5_ probe we have identified the karyotypes of 394 tadpoles total. Number of analyzed tadpoles in different clutches varied from 100% of surviving tadpoles (i.e. all surviving tadpoles were analyzed) in crosses 1_2013, 3_2013, 5_2013, 13_2014 to approximately 25% (i.e. about quarter of all obtained tadpoles were analyzed) in crosses 4_2013, 2_2013. We analyzed genomes transmitted in growing oocytes of 3 diploid and 9 triploid hybrid females used in the crossing experiments (Figs. 1, 2). Additionally, we have identified the genome composition of the oocytes in 3 diploid hybrid females used in crossing experiments and 3 triploid hybrid females that were not used in the crossings (Additional file 3: S1). Lampbrush chromosomes were identified according to earlier constructed cytological maps for both parental species (*P. ridibundus* and *P. lessonae*) [25, 28].

### Triploid hybrids produced diploid hybrids and parental species individuals in the R-E system

In order to estimate the role of triploid hybrids in the maintenance of studied population systems we have performed crossings using triploid females. Crossings of hybrid females with RRL genotype and hybrid males with RL genotype gave rise to tadpoles that were subdivided into three groups by FISH karyotyping: three clutches with only *P. ridibundus* tadpoles*,* a clutch with only *P. esculentus* tadpoles and three clutches with both *P. ridibundus* and *P. esculentus* tadpoles (Fig. 1, 3a, b). Crosses of a triploid female with RRL genotype and *P. ridibundus* male produced only *P. ridibundus* tadpoles, as expected (Fig. 1). All hybrid females participating in the crossings produced oocytes with 13 bivalents corresponding to the bivalents of *P. ridibundus* as revealed by lampbrush chromosome analysis (Figs. 1, 4a, b; Additional file 4: Figure S2a, a`). For almost all triploid hybrid females with RRL genotypes we analyzed from 13 to 28 full chromosomal sets, except for one female (f_16_2014) where only 5 full chromosomal sets were analyzed. Furthermore, we have analyzed 45 full lampbrush chromosomal sets from three additional triploid females with RRL genotype (f_10_2014, f_11_2014, f_4_2014 in Additional file 1: Table S1) that were not used in the crossing experiments. We have discovered that they produced oocytes with 13 bivalents of *P. ridibundus* chromosomes (Additional file 3: Figure S1). The results correspond to previously published data on lampbrush chromosomes obtained from the oocytes of the triploid females from the same population systems (see [25]). We conclude that triploid frogs with RRL genotype give rise to diploid animals due to formation of haploid gametes with the *P. ridibundus* genome (Fig. 1). Earlier studies on di- and triploid hybrid frogs from L-E and E systems have revealed similar contribution of triploid hybrids to the appearance of diploid animals [7, 13, 14, 16–20].

**Fig. 3:**
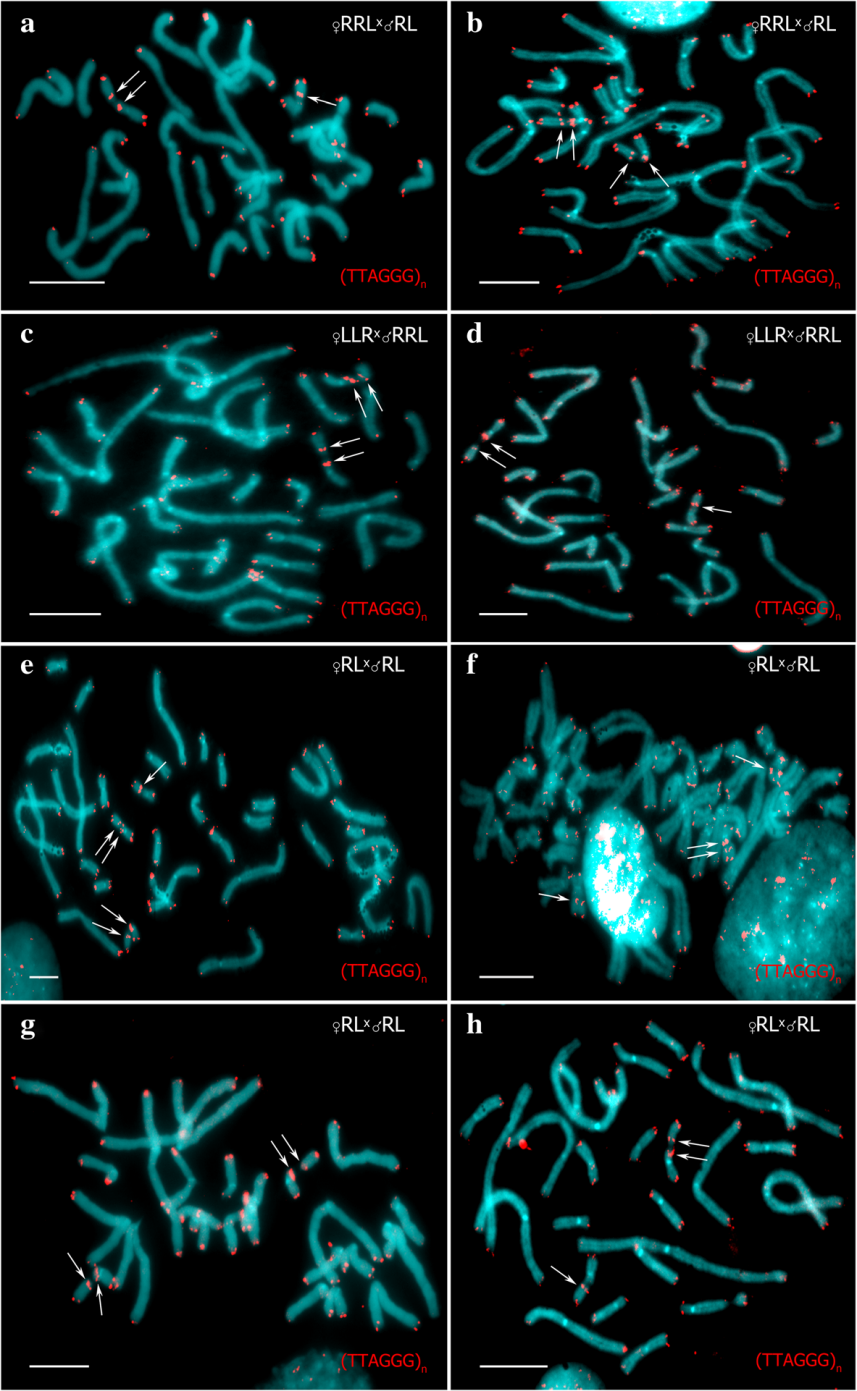
FISH mapping of interstitial (TTAGGG)_n_ repeat sites allows to identify karyotypes of tadpoles resulting from artificial crosses of hybrid animals. Metaphase chromosomes of tadpoles after FISH with (TTAGGG)_5_. One or two interstitial (TTAGGG)_n_ repeat sites (indicated by arrows) are distinguished in parental NOR-bearing chromosomes. Karyotypes of tadpoles resulting from crosses of triploid hybrid female with RRL genotype and diploid hybrid male (**a**, **b**), triploid hybrid female with LLR genotype and triploid hybrid male with RRL genotype (**c**, **d**) and two diploid hybrid frogs (**e**, **f**, **g**, **h**). Tadpoles were identified as *P. ridibundus* (**b**, **c**, **g**), diploid hybrids (**a**, **d**, **h**) and triploid hybrids with RRL (**e**) and LLR (**f**) genotypes. Scale bars = 10 μm

**Fig. 4:**
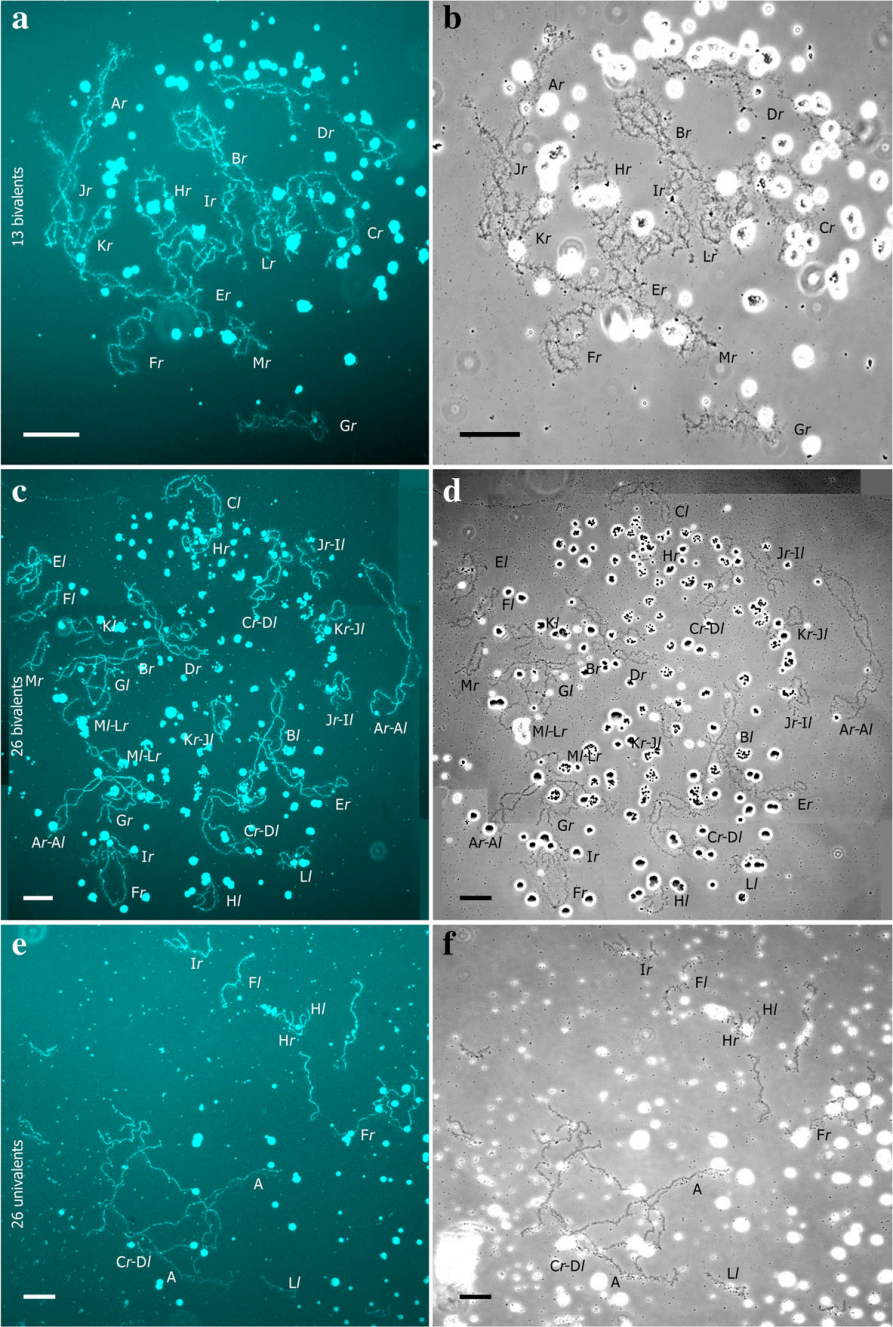
Lampbrush chromosome sets from growing oocytes of diploid and triploid hybrid frog with RRL genotype. Lampbrush chromosome sets from oocytes of triploid hybrid frog with RRL genotype represented by 13 bivalents corresponding to chromosomes of *P.ridibundus* (**a**, **b**). Lampbrush chromosome sets from oocytes of diploid hybrid frog represented by 26 bivalents (**c**, **d**) and 26 univalents (**e**, **f**) with 13 bi- or univalents corresponding to *P. ridibundus* chromosomes and 13 bi- univalents corresponding to *P. lessonae* chromosomes. Lampbrush chromosomes are numbered alphabetically; italic type indicates correspondence of the identified chromosome to genotype of parental species: *l* – to *P. lessonae*, *r* – to *P. ridibundus*. Chromosomes were counterstained with DAPI (**a**, **c**, **e**). Corresponding phase-contrast micrographs are shown (**b**, **d**, **f**). Scale bars = 50 μm

A crossing of triploid hybrid female with LLR genome composition and triploid male with RRL genome composition produced 33 *P. ridibundus* and one *P. esculentus* tadpoles. Contrary to crossing results, all analyzed oocytes (6 oocytes) from such females contained 13 bivalents corresponding to *P. lessonae* chromosomes (Fig. 1). Mismatch between crossing results and lampbrush chromosome analysis may be associated with higher rate of the *P. lessonae* tadpoles mortality compared to the *P. ridibundus* tadpoles, as overall survival of tadpoes from this clutch was low (Additional file 2: Table 2). We propose that the triploid LLR female produced gametes with *P. ridibundus* genome and gametes with *P. lessonae* genome but different survival rates of *P. esculentus* and *P. ridibundus* tadpoles skew gamete contribution towards *P. ridibundus*. Previous studies have demonstrated that triploid hybrids with LLR genotype frequently demonstrate deviations in gametogenesis sometimes leading to unusual gametes with *P. ridibundus* genome [25, 43]. Higher levels of deviations during gametogenesis in the LLR hybrids compared to the RRL hybrids were also observed in other population systems. Triploids with LLR genotype usually form haploid gametes with *P. lessonae* genome after elimination of single-copy *P. ridibundus* genome during gametogenesis, however LL and RL gametes can be occasionally produced as well [7, 17, 19, 20]. We suggest that triploid hybrid male with RRL genotype produced gametes with chromosomes of *P. ridibundus,* this being in agreement with earlier results of studies on triploid males in various population systems [7, 13, 16, 18–20].

We conclude that oocytes with 13 bivalents generated by the triploid hybrid *P. esculentus* females can produce haploid gametes and give rise to the diploid *P. ridibundus* or *P. esculentus* animals.

### Diploid hybrids contribute to appearance of triploid and diploid hybrids in the R-E system

In order to estimate the role of diploid hybrids in the maintenance of R-E system studied we have carried out crossings of the diploid hybrid frogs with each other and *P. ridibundus* males. In one crossing of two diploid hybrids, triploid tadpoles with RRL and LLR genotypes appeared (Figs. 2, 3e, f). Lampbrush chromosome analysis of 41 oocytes with full chromosomal sets obtained from this hybrid female revealed that 15 oocytes contained 26 bivalents and 10 oocytes contained 26 univalents. In these oocytes 13 bivalents or univalents corresponded to *P. ridibundus* lampbrush chromosomes and the other 13 bivalents or univalents corresponded to *P. lessonae* lampbrush chromosomes. In the other crossing of diploid hybrid female and diploid hybrid male, we observed not only tadpoles with RRL and LLR genotypes but also diploid *P. esculentus* and *P. ridibundus* tadpoles (Figs. 2, 3g, h). Lampbrush chromosome  analysis demonstrated that in this female 18 oocytes contained 13 *P. ridibundus* bivalents, 19 oocytes contained 26 bivalents and 7 oocytes contained 26 univalents (Figs. 2, 4c-f; Additional file 4: S2b-d`). In oocytes with 26 bivalents and univalents 13 bi- or univalents corresponded to *P. ridibundus* lampbrush chromosomes and the other 13 bi- or univalents corresponded to *P. lessonae* lampbrush chromosomes. We suggest that oocytes with 26 univalents cannot complete meiotic division successfully and result in aneuploid gametes. The oocytes with 26 bivalents can overcome this problem and form diploid RL gametes with both *P. ridibundus* and *P. lessonae* genomes (Fig. 2). Fertilization of such gametes gives rise to triploid hybrids. We have also analyzed oocyte genome composition of the three diploid females that were not used in the crossing experiments (f_12_2014, f_5_2014, f_9_2014 in Additional file 1: Table S1). For two diploid hybrid females we have analysed 15 and 16 full lumpbrush chromosomal sets and have found that all oocytes contained *P. ridibundus* chromosomes. After analysis of 7 lampbrush chromosomal sets of diploid hybrid females we found oocytes with 13 bivalents corresponding to *P. ridibundus* chromosomes, oocytes with 26 bivalents and oocytes with 26 univalents, where 13 bi- or univalents corresponded to the chromosomes of *P. ridibundus* and the other 13 bi- or univalents corresponded to the chromosomes of *P. lessonae* (Additional file 3: Figure 1). In the majority of other known L-E and E systems triploid hybrid frogs are thought to emerge after fertilization of diploid gametes [7, 14, 17–20, 44].

In the studied clutches of diploid hybrid females, eggs were larger and more varied in size compared to clutches of triploid hybrids. These dissimilarities in size remained during tadpoles development up to the end of the metamorphosis. Individual hybrid females often produce eggs with variable size, this has previously been considered to be a sign of different ploidy [14, 26, 27, 44]. Here, we have, for the first time, identified the karyotypes of the tadpoles developing from such eggs and compared the data with direct observation of chromosome sets in growing oocytes. Our analysis has revealed that tadpoles resulting from crosses of females that produce variable oocytes differed in ploidy and genome composition. We found that oocytes with 26 bivalents and 13 bivalents produced by the individual diploid hybrid females were able to proceed past meiosis stage and give rise to viable progeny with different genome compositions and ploidy (Fig. 2; Additional file 3: Figure 1; see [25]).

After crossing of one diploid RL female with *P. ridibundus* male we have obtained *P. ridibundus* and diploid *P. esculentus* tadpoles (Fig. 2). Analysis of lampbrush chromosomes unexpectedly showed that 25 oocytes of this particular female posessed 26 bivalents and 6 oocytes comprised 26 univalents where 13 bivalents or 13 univalents corresponded to *P. ridibundus* chromosomes and other 13 bivalents or 13 univalents corresponded to *P. lessonae* chromosomes. After meiosis, the oocytes with 26 bivalents should produce diploid gametes which after fertilization by sperm with *P. ridibundus* genome should give rise to triploid tadpoles. Nevertheless, our crossing results do not correspond to the expected way of egg formation from oocytes with 26 bivalents. We can only speculate that this diploid female either represents a rare example of some uncommon way of oogenesis, or possess strong mosaicism of germ cells.

Karyotype analysis of tadpoles resulting from crossing experiments allowed us to estimate contribution of *P. esculentus* males to the maintenance of the studied R-E systems. We propose that diploid males producing sperm with *P. lessonae* genome participate in the appearance of the majority of diploid hybrid frogs and triploid frogs with LLR genotype (Figs. 1, 2). We can conclude that four diploid males produced haploid gametes with only *P. ridibundus* genome, one male produced haploid gametes with *P. lessonae* genome, and five males produced a portion of haploid gametes with *P. ridibundus* genome and a portion of haploid gametes with *P. lessonae* genome (Figs. 1, 2). Similar results for the population systems under investigation were obtained after detailed analysis of the hybrid male cells using DNA flow cytometry [43]. Biryk and co-authors found that majority of diploid males produced mixed sperm represented by a portion of sperm with *P. lessonae* genome and a portion of sperm with *P. ridibundus* genome [43]. Other diploid hybrid males produced haploid sperm only with *P. ridibundus* genome; while occasional males produced haploid sperm with *P. lessonae* genome [43]. The phenomenon where males simultaneously produce haploid gametes with *P. ridibundus* genome and haploid gametes with *P. lessonae* genome was earlier observed in diploid hybrid males and called hybrid amphispermy [30]. Such males were occasionally found in the other population systems but their relative abundance in the population systems and important role in the appearance of diploid hybrids was not previously reported [7, 17, 19, 20].

### Micronuclei are abundant in gonads of *P. esculentus* but not *P. ridibundus* tadpoles

To assess the presence of germ cells in gonads of hybrid tadpoles and to observe genome elimination at different stages of gonad development we have performed morphological analysis of 32 gonads from the *P. ridibundus* tadpoles and 40 gonads from the hybrid tadpoles. 15 *P. ridibundus* tadpoles were analyzed from crossing of two *P. ridibundus* individuals (cross 4_2013) and additionally 17 *P. ridibundus* tadpoles were analyzed from crosses of triploid *P. esculentus* with RRL genotype with diploid hybrid male (crosses 8_2014, 2_2013). We have not found any differences in germ cell distribution between *P. ridibundus* tadpoles from various crosses. *P. ridibundus* and *P. esculentus* tadpoles after stage 30 of development were designated as females according to classification suggested by Ogielska, Kotusz [45] and Haczkiewicz, Ogielska [46]. We have observed morphologically different developmental stages of gonads in *P. ridibundus* tadpoles: stage before sexual differentiation (stages 25–27 according to Gosner [40]; stages of gonads development 1–3 according to Ogielska, Kotusz [45]), stage of sexual differentiation with high mitotic activity (stages 28–34 according to Gosner [40]; stages of gonads development 4 according to Ogielska, Kotusz [45]), stages of meiocyte and diplotene oocyte formation (stages 35–41 according to Gosner [40]; stages of gonads development 5–9 according to Ogielska, Kotusz [45]) (Fig. 6a, b). Germ cells were clearly distinguishable and were represented by large cells with faint chromatin staining including large amount of intranuclear bodies such as amplified nucleoli and coilin-positive bodies (Fig. 6 a, b).

Germ cells were present in all gonads dissected from 18 diploid and 22 triploid hybrid tadpoles from five different crossings (crossings 14_2014, 6_2014, 5_2013, 3_2013, 3_2014) (Figs. 1, 2, 6c-e). Lower amount of germ cells compared to other hybrid tadpoles was observed in two hybrid tadpoles (from crosses 5_2013 and 14_2014). In gonads of all hybrid tadpoles, we have observed DAPI-positive bodies (micronuclei or “nucleus like bodies” according to Ogielska [38]) (Fig. 6c-g). Number of micronuclei varied from one to five per individual germ cell. The majority of micronuclei had more intensive DAPI staining, indicating condensed chromatin state compared to the interphase nuclei (Fig. 6c-g). The micronuclei were found in 35% of germ cells during hybrid tadpoles development at stages 27–29, in 30% of germ cells during hybrid tadpoles development at stages 30–33 and in 15% of germ cells during hybrid tadpoles development at stages 34–36. Gonads of *P. ridibundus* tadpoles lacked micronuclei at the same developmental stages (Fig. 6a, b), which corresponds to results obtained by Ogielska [38].

**Fig. 5:**
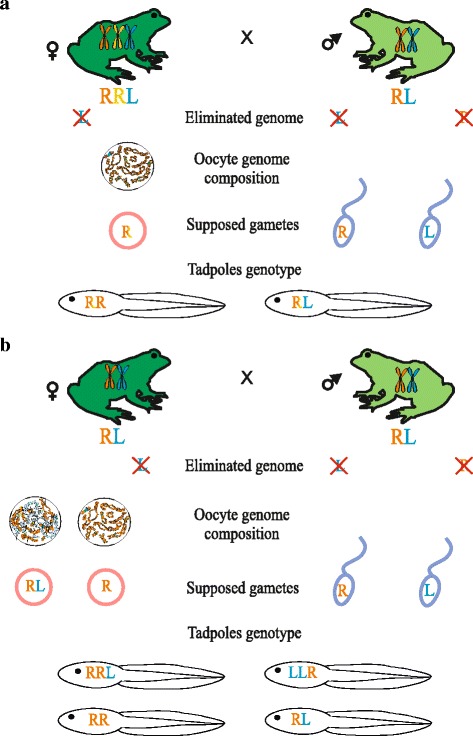
Contribution of diploid and triploid hybrid frogs in maintenance of studied R-E systems. **a** Crossings of triploid hybrid females with RRL genotype and diploid hybrid males led to appearance of *P. ridibundus* and diploid *P. esculentus* tadpoles. Triploid hybrid females produced haploid eggs with *P. ridibundus* genome while diploid hybrid males produced haploid sperm with either *P. ridibundus* or *P. lessonae* genomes. **b** Crossings of two diploid hybrid frogs led to appearance of triploid tadpoles with RRL and LLR genotypes, *P. ridibundus* tadpoles and diploid *P. esculentus* tadpoles. Diploid hybrid females produced diploid eggs with both *P. ridibundus* and *P. lessonae* genomes, and haploid eggs with *P. ridibundus* genome. Diploid hybrid males presumably produced haploid sperm with either *P. ridibundus* or *P. lessonae* genomes

**Fig. 6:**
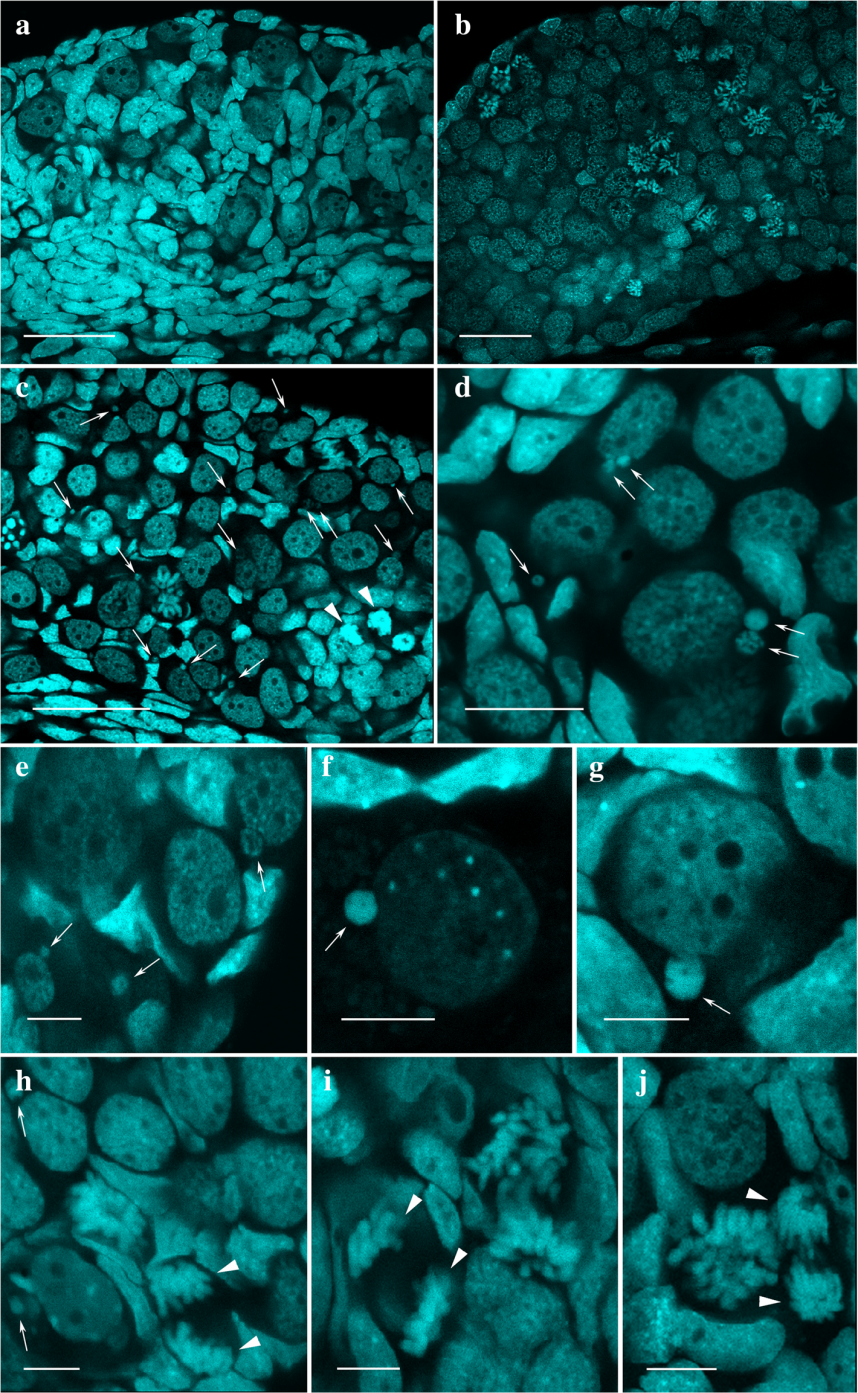
Detection of micronuclei in gonads of hybrid tadpoles. Morphological analysis of gonads dissected from *P. ridibundus* (**a**, **b**) tadpoles, diploid (**e**, **g**, **h**) and triploid (**c**, **d**, **f**, **i**, **j**) *P. esculentus* tadpoles. Undifferentiated gonads (stage 3) from *P. ridibundus* tadpoles (**a**). Gonads at stage of high mitotic activity (stage 4) from *P. ridibundus* tadpoles (**b**). Scale bars = 50 μm. Micronuclei (arrows) were abundant in cytoplasm of germ cells in gonads from diploid (**e**, **g**, **h**) and triploid (**c**, **d**, **f**, **i**, **j**) hybrid tadpoles. Micronuclei vary in size and chromatin density. Anaphase stage of mitosis (**h**, **i**, **j**); no lagging chromosomes (arrowheads) was observed. Scale bar for c = 50 μm; d = 25 μm; e-j = 10 μm

The results allow us to confirm the suggestion of Ogielska [38] that micronuclei contain eliminated genome of *P. lessonae* (Figs. 1, 2, 5). Nevertheless, precise genome identification is further required. Two alternative mechanisms may lead to appearance of micronuclei in the cytoplasm of germ cells: budding from interphase nucleus [38, 47] and after lagging of chromosomes which are then independently surrounded by nuclear membrane [38, 47]. Nevertheless, in gonad cells of all examined hybrid tadpoles we did not observe any instances of chromosome lagging in 31 anaphase and telophase cells analyzed (Fig. 6h-j). Thus, we propose that chromatin budding from interphase nucleus as the more plausible mechanism of selective genome elimination.

### On the universal mechanism of maintenance of the hybrid frogs population systems

In the studied R-E systems, the majority of triploid females with RRL genotype, some diploid hybrid females and even some triploid females with LLR genotype unexpectedly produced haploid gametes with *P. ridibundus* genome, although hybrid frogs co-exist with *P. ridibundus* individuals ([25, 43] and data presented here). Both forms of triploid hybrid frogs cannot propagate themselves and depend on the diploid eggs produced by some diploid females (Figs. 1, 2, 5a, b). Our earlier results allow us to propose that some triploid females with LLR genotype are also able to produce diploid eggs [25]. Triploid hybrid frogs with RRL genome composition can potentially replace *P. ridibundus* individuals in the population from the reproduction perspective through production of haploid gametes with *P. ridibundus* genome (Figs. 1, 2, 5a, b). The *P. ridibundus* individuals can regularly re-appear in the studied population systems because of large amounts of male and female gametes with *P. ridibundus* genome (Figs. 1, 2, 5a, b).

Artificial crossings experiments have allowed for significant insights into the mechanisms of population maintenance for L-E and E systems [7, 11, 17, 20, 44]. The contribution of hybrid animals to the maintenance of R-E systems in Poland and Germany was earlier established by experimental crossings experiments [13, 15, 16]. Nevertheless, the R-E system investigated previously included only *P. ridibundus* females that co-existed either with diploid hybrid males or triploid hybrid males of the LLR genotype [15, 48]. Here, we present data on the R-E system which is characterized by a more complex structure of hybrid forms and by this parameter seems to be similar to the L-E and E systems [22, 43]. In a recent study Ragghianti and co-authors [49] made use of four hybrid males from complex R-E systems from Poland. The authors show that hybrid males produce sperm with either *P. ridibundus* or *P. lessonae* genomes being similar to our results [49]. Unfortunately, no females or triploid animals from the R-E systems from Poland were included in that study.

In the majority of the known hybrid complexes, including fish and amphibians, triploid hybrids reproduce via clonal or hemiclonal reproductive modes such as gynogenesis or kleptogenesis [3, 4, 23]. Despite ability to gynogenic reproduction in some fish hybrid complexes, triploid hybrids can produce haploid gametes leading to diploids. At the same time diploid hybrid females are responsible for the maintenance of triploids in complex population systems [3, 4, 24, 50]. During evolution process, various amphibian and fish hybrid complexes independently developed similar mechanisms leading to appearance and maintenance of diploid and triploid hybrids when they co-exist in the population system.

## Conclusions

Based on results presented we propose a model of diploid and triploid hybrid frogs reproduction in a population system containing both hybrids and *P. ridibundus* individuals. Triploid hybrids with RRL genotype produce oocytes with 13 bivalents which form haploid gametes with *P. ridibundus* genome. After fertilization of such gametes diploid hybrids and *P. ridibundus* individuals appear. Thus, triploid hybrids with RRL genotype can potentially aid in maintaining *P. ridibundus* population they co-exist with because they produce haploid gametes with the same genome composition. Diploid hybrid females also typically produced haploid gametes with *P. ridibundus* genome. We note the unusual situation in which diploid and triploid hybrid females produced gametes with the same genome as the parental species hybrids co-exist with. Diploid hybrids as well as triploid hybrids with LLR genome composition appeared after fertilization by sperm with *P. lessonae* genome which is produced by some of the diploid hybrid males. Moreover, the diploid hybrid females can produce oocytes with 26 bivalents corresponding to chromosomes of both parental species. Such oocytes lead to diploid gametes which give rise to triploid progeny after fertilization. The majority of the observed diploid and all observed triploid hybrid tadpoles resulting from crossing experiments show similar amount of germ cells that is found in parental species. In the gonads of hybrid tadpoles we have observed micronuclei in the cytoplasm of germ cells that may indicate genome elimination. Thus, triploid hybrids cannot reproduce and maintain themselves in population systems without diploid hybrid females producing diploid eggs.

## Additional files

Additional file 1: Table S1. List of studied European water frogs from the R-E systems in Eastern Ukraine. (XLSX 18 kb)

Additional file 2: Table S2. Survival rate of tadpoles during early developmental stages. (XLSX 10 kb)

Additional file 3: Figure S1. Lampbrush chromosome analysis of additional diploid and triploid hybrid frogs which did not participated in crossings. Analysis of lampbrush chromosomes obtained from growing oocytes show that diploid hybrid females produced oocytes with 26 bi- and univalents corresponding to *P. ridibundus* (orange) and *P. lessonae* (blue) chromosomes and 13 bivalents corresponding to *P. ridibundus* (orange) chromosomes. Triploid hybrid females produced oocytes with 13 bivalents corresponding to *P. ridibundus* chromosomes (orange). (PDF 2058 kb)

Additional file 4: Figure S2. Identification of individual lampbrush chromosomes using FISH mapping of interstitial (TTAGGG)_n_ repeat sites. Lampbrush chromosomes from oocytes of triploid (a, a`) and diploid (b,b`, c,c`) hybrid frog. a,a` Bivalent consisting of lampbrush chromosomes that correspond to *P. ridibundus* chromosome H (from full lampbrush chromosomal set with 13 bivalents of *P. ridibundus* depicted in Fig. 3a, b). b,b`,c,c` Bivalents consisting of lampbrush chromosomes that correspond to *P. ridibundus* and *P. lessonae* lampbrush chromosomes (from full lampbrush chromosomal set with 26 bivalents depicted in Fig. 4c, d). d,d` Univalents corresponding to *P. ridibundus* and *P. lessonae* lampbrush chromosomes (from full lampbrush chromosomal set with 26 univalents depicted in Fig. 4e, f). Interstitial (TTAGGG)_n_ repeat sites are shown by square brackets. Chromosomes were counterstained with DAPI. Arrowheads show centromeres. Arrows indicate the most prominent marker loops. Scale bars = 10 μm. (PDF 12710 kb)

## Abbreviations

*DAPI*: 4′,6-diamidino-2-phenylindole
*FISH*: Fluorescence in situ hybridization
*MS 222*: 3-aminobenzoic acid ethyl ester
*NOR*: Nucleolus organizer region
*PBS*: Phosphate buffered saline
*SSC*: Saline-sodium citrate buffer
