# Supplementary material for: Mutual maintenance of di- and triploid Pelophylax esculentus hybrids in R-E systems: results from artificial crossings experiments
Source: BMC Evol Biol. 2017 Oct 17;17:220. doi: 10.1186/s12862-017-1063-3 (PMC5645918; doi:10.1186/s12862-017-1063-3)
Supplement: Supplementary file 3 — Lampbrush chromosome analysis of additional diploid and triploid hybrid frogs which did not participated in crossings. Analysis of lampbrush chromosomes obtained from growing oocytes show that diploid hybrid females produced oocytes with 26 bi- and univalents corresponding to P. ridibundus (orange) and P. lessonae (blue) chromosomes and 13 bivalents corresponding to P. ridibundus (orange) chromosomes. Triploid hybrid females produced oocytes with 13 bivalents corresponding to P. ridibundus chromosomes (orange). (PDF 2058 kb) [file 12862_2017_1063_MOESM3_ESM.pdf]

crossings number	females genotype	number of analyzed oocytes	oocyte genome composition	
f_9_2014	♀RL	16	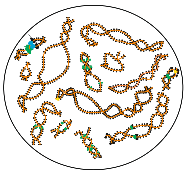	13 bivalents of *P. ridibundus*
f_12_2014	♀RL	15	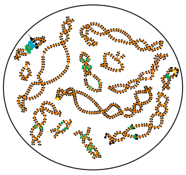	13 bivalents of *P. ridibundus*
f_5_2014	♀RL	3	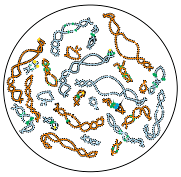	26 bivalents of *P. ridibundus* and *P. lessonae*
		2	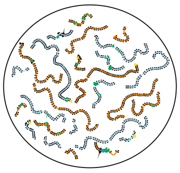	26 univalents of *P. ridibundus* and *P. lessonae*
		2	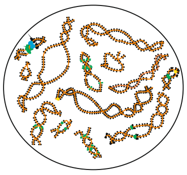	13 bivalents of *P. ridibundus*
f_4_2014	♀RRL	16	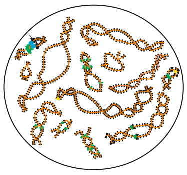	13 bivalents of *P. ridibundus*
f_10_2014	♀RRL	17	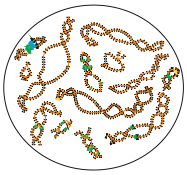	13 bivalents of *P. ridibundus*
f_11_2014	♀RRL	12	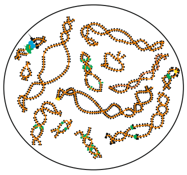	13 bivalents of *P. ridibundus*
