# Supplementary figures and images for: Mutual maintenance of di- and triploid Pelophylax esculentus hybrids in R-E systems: results from artificial crossings experiments
Source: BMC Evol Biol. 2017 Oct 17;17:220. doi: 10.1186/s12862-017-1063-3 (PMC5645918; doi:10.1186/s12862-017-1063-3)

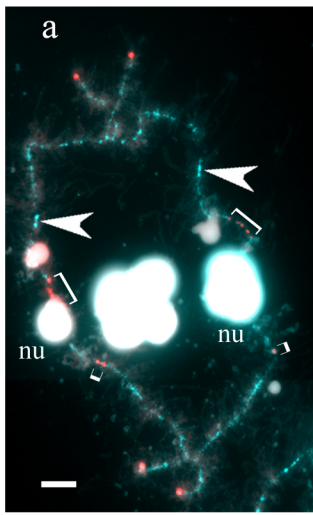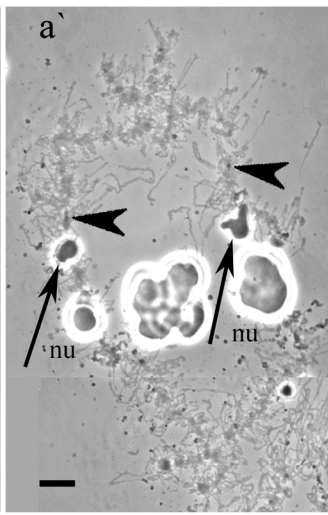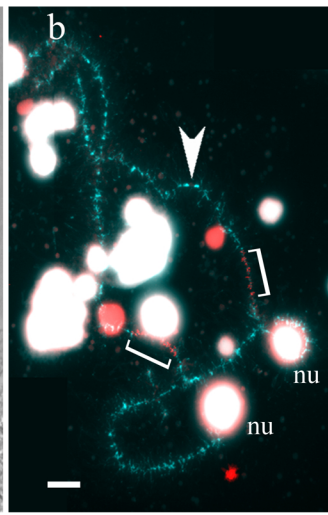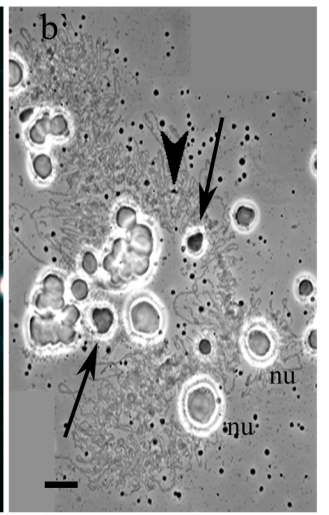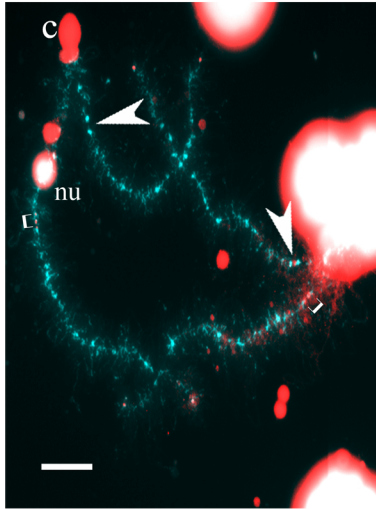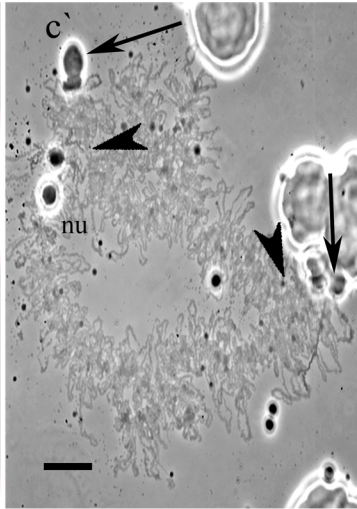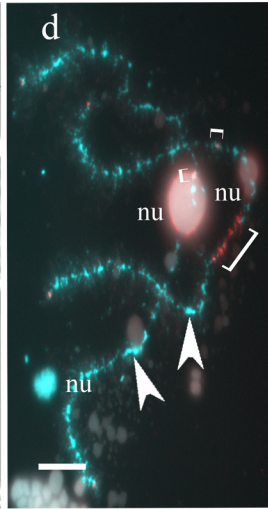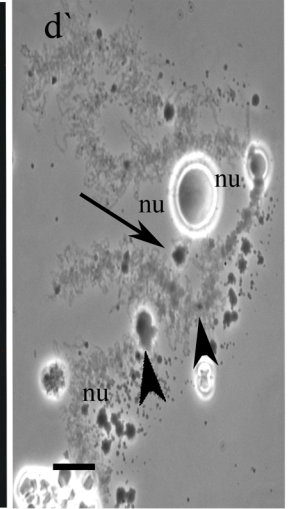

Supplement: Supplementary file 4 — Identification of individual lampbrush chromosomes using FISH mapping of interstitial (TTAGGG)n repeat sites. Lampbrush chromosomes from oocytes of triploid (a, a`) and diploid (b,b`, c,c`) hybrid frog. a,a` Bivalent consisting of lampbrush chromosomes that correspond to P. ridibundus chromosome H (from full lampbrush chromosomal set with 13 bivalents of P. ridibundus depicted in Fig. 3a, b). b,b`,c,c` Bivalents consisting of lampbrush chromosomes that correspond to P. ridibundus and P. lessonae lampbrush chromosomes (from full lampbrush chromosomal set with 26 bivalents depicted in Fig. 4c, d). d,d` Univalents corresponding to P. ridibundus and P. lessonae lampbrush chromosomes (from full lampbrush chromosomal set with 26 univalents depicted in Fig. 4e, f). Interstitial (TTAGGG)n repeat sites are shown by square brackets. Chromosomes were counterstained with DAPI. Arrowheads show centromeres. Arrows indicate the most prominent marker loops. Scale bars = 10 μm. (PDF 12710 kb) [file 12862_2017_1063_MOESM4_ESM.pdf]
